# Unexpected behavioural adaptation of yellow fever mosquitoes in response to high temperatures

**DOI:** 10.1038/s41598-024-54374-5

**Published:** 2024-02-13

**Authors:** David O. H. Hug, Alida Kropf, Marine O. Amann, Jacob C. Koella, Niels O. Verhulst

**Affiliations:** 1 National Centre for Vector Entomology, Institute of Parasitology, Vetsuisse and Medical Faculty, University of Zürich, Zurich, Switzerland; 2 Laboratory of Ecology and Epidemiology of Parasites, Institute of Biology, University of Neuchâtel, Neuchâtel, Switzerland

**Keywords:** Evolutionary ecology, Ecology, Climate sciences, Experimental evolution

## Abstract

Temperature is a major ecological driver of mosquito-borne diseases as it influences the life-history of both the mosquito and the pathogen harboured within it. Understanding the mosquitoes’ thermal biology is essential to inform risk prediction models of such diseases. Mosquitoes can respond to temperatures by microhabitat selection through thermal preference. However, it has not yet been considered that mosquitoes are likely to adapt to changing temperatures, for example during climate change, and alter their preference over evolutionary time. We investigated this by rearing six cohorts of the yellow fever mosquito *Aedes aegypti* at two temperatures (24 °C, 30 °C) for 20 generations and used these cohorts to explicitly separate the effects of long-term evolution and within-generation acclimation on their thermal preferences in a thermal gradient of 20–35 °C. We found that warm-evolved mosquitoes spent 31.5% less time at high temperatures, which affects their efficiency as a vector. This study reveals the complex interplay of experimental evolution, rearing temperatures, and thermal preference in *Ae. aegypti* mosquitoes. It highlights the significance of incorporating mosquito microhabitat selection in disease transmission models, especially in the context of climate change.

## Introduction

Rising global mean temperatures have significant implications for the epidemiology of vector-borne diseases as they can lead to alterations in disease prevalence and geographic distribution^1^. This is partly due to the considerable influence of temperature on both vectors and pathogens harboured within them. Indeed, temperature is one of the most important environmental factors driving the dynamics of vector-borne transmission. It moulds the physiology, life cycles, behaviours, and disease transmission competences of their vectors, notably mosquitoes^2–13^. Mosquitoes are major vectors of several pathogens including dengue virus, West Nile virus, *Plasmodium spp.* (the causative agent of malaria), and diverse filarial nematodes. This collective burden of pathogens takes a toll on both human and animal health, imposing challenges upon healthcare professionals and veterinary practitioners^2^.

Climate change, driven by global temperature shifts, has emerged as a critical driver influencing the transmission dynamics of these vector-borne diseases^14^. Understanding the thermal behaviour and thermal adaptation of mosquito species is thus important.

Research has demonstrated that mosquitoes exhibit specific thermal preferences across laboratory, semi-field, and field systems^15,16^. Notably, many mosquito species exhibit a preference for temperatures cooler than the mean ambient temperature, which is believed to be an adaptive response to mitigate heat stress in their natural breeding habitats^15–19^.

In contrast to the extensive research on phenotypic temperature preferences in mosquitoes, the mechanisms underlying genetic or plastic adaptations in their thermal preferences remain relatively unexplored^20^. Model organisms like *Drosophila* flies, characterized by short life cycles and large populations, have been employed to investigate evolutionary changes in thermal preference over multiple generations^21,22^. In *Drosophila melanogaster*, significant changes in food-seeking behaviour, physiological characteristics, and temperature preferences were observed. After evolution at higher rearing temperatures in a range of 27 to 30 °C for over 15 generations flies were not just significantly smaller than the ones reared at colder temperatures, in addition they showed more intense food-searching behaviour and preferred higher temperatures^22^. Furthermore, field studies conducted in eastern coastal Australia^23^ revealed adaptations in response to changing temperature and humidity levels in *Drosophila melanogaster* populations spanning a period of 20 years.

Very little is known about adaptative changes in thermal preference in mosquitoes. A study dating back to 1938, focusing on *Culex quinquefasciatus*, examined the thermal behaviour under rearing conditions with an adaptation period of only one generation. Despite a tendency to avoid high temperatures, no discernible changes in behaviour or avoidance of high or low temperatures were observed when mosquitoes were acclimated to a temperature of 30 °C^24^.

*Aedes aegypti* is an ideal candidate for such artificial selection or experimental evolution experiments due to its diminutive size and fast developmental time, like other small ectotherms e.g., *D. melanogaster*^25^. Our study focuses on the capacity of *Ae. aegypti* mosquitoes to adapt their behaviour to changing temperatures. Through selective breeding over 20 generations at two different temperatures (24 °C and 30 °C), we aimed to investigate how thermal preferences evolve in this species. In addition to experimental evolution over 20 generations we also investigated the effect of acclimation in the last generation by cross-temperature rearing adapted mosquito cohorts from egg to adult at both 24 °C and 30 °C. With the underlaying assumption that mosquitoes can choose a temperature to maximise their performance and fitness, we expect this temperature to change with thermal adaptation and acclimation. Thus, it would be beneficial to evolve mechanisms to cope with increasing temperatures. In addition, we also expect the thermal preference to change since better heat tolerances should also increase the preference for temperatures that increase fitness.

## Results

In total 1024 individual mosquitoes were released in a thermal gradient setup (20–35 ± 0.5 °C) in cohorts of 12 and were able to select their preferred temperature. The insects were tracked by video analysis software like previously described^15^ and their behaviour categorized in flying, walking and resting. Overall mean mortality during acclimation was about 24% and did not differ between acclimation (24 °C or 30 °C for one generation) temperatures (LM, F_1_ = 0.41, *P* = 0.525) or experimental evolution (24 °C or 30 °C for 20 generations) temperature (LM, F_1_ = 1.50, *P* = 0.225). Mosquitoes could be video tracked 91.7% of the time spent in the arena. However, mosquitoes acclimated to 24 °C could be tracked 4% longer than mosquitoes acclimated to 30 °C regardless of their evolutionary temperature (24 °C: 93.5% vs. 30 °C: 89.75%).

Since the three cohorts within temperatures did not differ from each other overall, they were combined in the analysis (post-hoc, all *P* > 0.084). A full overview of the statistics can be found in supplementary Table S1 and S2.

### Experimental evolution

**Figure 1:**
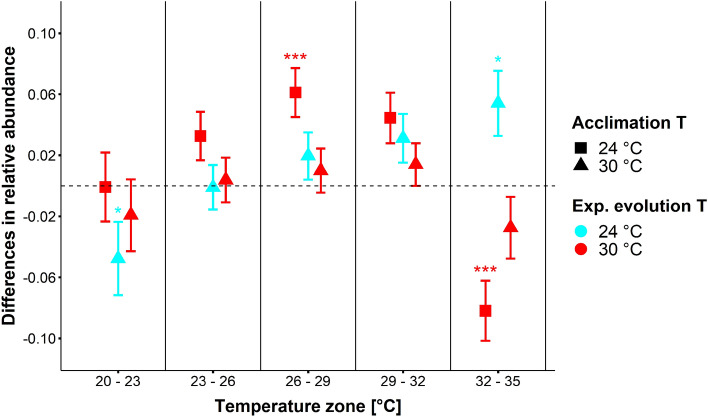
Effect of experimental evolution and acclimation on thermal preferences. Shown are differences of mean relative abundance ± standard errors of each treatment from the base-line abundance (evolved 24 °C, acclimated 24 °C) in the five temperature zones (20–23 °C, 23–26 °C, 26–29 °C, 29–32 °C, 32–35 °C) for mosquitoes evolved at 24 °C (cyan) vs. 30 °C (red) and acclimated at 24 °C (rectangle) and 30 °C (triangle) for all movement types (flying, walking, and resting) combined. Data points represent the difference from the baseline in mean relative abundance in given zone of all mosquitoes of a given temperature treatment. Significant (* = *P* < 0.05, ** = *P* < 0.01, *** = *P* < 0.001) differences refer to treatments within one zone (Tukey; 95% CI). N = 527 acclimated at 24 °C; 497 at 30 °C; 505 evolved at 24 °C and 519 at 30 °C.

Considering all behavioural types together (cumulative duration, supplementary Fig. S1), there was a long-term effect of experimental evolution on the presence of a mosquito in a given temperature zone (generalized linear mixed effect model [GLMM], Chi^2^_4_ = 30.01, *P* < 0.001). Mosquitoes that evolved at the higher temperature (30 °C) spent 31.5% less time in the warmest zone than those evolved at 24 °C (GLMM, Chi^2^_4_ = 57.61, *P* < 0.001) regardless of the temperature at which they were acclimated to. This result remained the same when separating each evolutionary temperature group according to their acclimation temperatures (each post-hoc *P* < 0.001; Fig. 1). The experimental day had an additional significant effect when included in the model (GLMM, Chi^2^_4_ = 27.10, *P* = 0.034).

When splitting movement types into flying, walking, and resting, differences between movement types appeared (Fig. S2, S3 and S4). Flying mosquitoes evolved at 30 °C spent less time in the warmest zone (Tukey, diff = 0.11, *P* < 0.001) than those evolved at 24 °C. The differences between temperature treatments were largest for flying mosquitoes (19.2%; GLMM_flying_, Chi^2^_4_ = 29.93, *P* < 0.001), intermediate for resting mosquitoes (13%; GLMM_resting_, Chi^2^_4_ = 15.27, *P* = 0.003), and smallest for walking mosquitoes (6%; GLMM_walking_, Chi^2^_4_ = 12.90, *P* = 0.012).

A reduction in mean velocity would indicate whether temperature impairs a mosquito’s activity (e.g., a ‘cold trap’ or “heat exhaustion”)^15,26^. Indeed, in the analysis with all movement types combined there were velocity differences between zones (linear model [LM], F_4_ = 13.93, *P* < 0.001). Velocity was lower in the coldest and warmest zones than in the other temperature zones (Tukey, *P* < 0.05). However, no differences between experimental evolution temperatures were found when the velocity of all movement types was combined.

### Acclimation

Mosquitoes that had been acclimated by being reared from larvae to adult in either of the two acclimation temperatures (24 °C or 30 °C) showed a trend opposite to that evident for the experimental evolution temperatures. When combining all movement types, mosquitoes acclimated to the warm temperature (30 °C) stayed in the warm zone 14.8% longer than the ones acclimated to the cooler temperature (24 °C; GLMM, Chi^2^_4_ = 42.10, *P* < 0.001). This result remained the same when separating each acclimation temperature group according to their evolutionary temperatures (post-hoc, each *P* < 0.001; Fig. 1). This was mainly caused by an increase in resting (12.9%; GLMM, Chi^2^_4_ = 21.86, *P* < 0.001; post-hoc, *P* < 0.001) and walking (10%; GLMM, Chi^2^_4_ = 20.68, *P* < 0.001; post-hoc, *P* < 0.001) times in the warm zone. No significant effect of acclimation temperature on flying time was found (GLMM, Chi^2^_4_ = 5.03, *P* = 0.284).

The velocity of walking was 5.8% higher for mosquitoes acclimated to the higher temperature (linear mixed effect model [LMM], Chi^2^_4_ = 24.63, *P* < 0.001, Fig. S3). Warmer-evolved mosquitoes were overall slower. Focusing on flying mosquitoes only, revealed differences between the two acclimation temperatures (LM, F_1_ = 48.00, *P* < 0.001, Fig. S2): the flying velocity of warm-acclimated mosquitoes was significantly lower (LM, t = − 2.25, *P* = 0.025). Mosquitoes also walked slower when they were warm-acclimated (LM, F_1_ = 7.95, *P* = 0.005) and a post-hoc-test of the interaction term (LM, F_4_ = 6.49, *P* < 0.001) revealed that in the warmest zone, warm-acclimated mosquitoes were significantly slower (Tukey, diff = 0.07, *P* < 0.001).

## Discussion

Our results support the hypothesis that *Ae. aegypti* mosquitoes that evolved at different temperatures changed their thermal preferences. In contrast to our expectations, rather than preferring the temperature in which they had evolved, mosquitoes that have evolved at the lower temperature (24 °C) preferred to spend more time in areas of higher temperature than mosquitoes that have evolved at the higher temperature (30 °C). The effect of evolution (31.5%) at high temperatures was about twice as strong as the effect of acclimation (14.8%).

### Evolved effect

Mosquitoes that evolved at higher temperature more strongly avoided the highest temperature zone and gathered more frequently in the coolest zone (Fig. 1).

An overall preference for cooler temperatures corroborates with the results reported in several recent studies for different mosquito species^15,16^ and other insects such as different *Drosophila* species^27^ and the yellow dung fly (*Scathophaga stercoraria*)^28^. In ectotherms, a preference for temperature below the thermal optimum can be explained by their thermal performance curves (TPC)^14,29,30^. Based on the metabolic theory of ecology, TPCs are usually characterized by a steeper decline in performance at temperature exceeding the thermal optimum which can have dramatic consequences on the fitness of ectotherms^31^. Choosing cooler microclimates can buffer insects from warm temperature extremes and thus contributes to reduced mortality^29^. In our experiment, this behaviour was only recorded in mosquitoes that evolved at warmer temperatures. A possible explanation for the lack of high temperature avoidance in mosquitoes that evolved at cooler temperature may be that they did not have to adapt to this dangerous range of high temperatures. This might be since they never encountered them in the first place and therefor show a restricted ability to detect and avoid them.

To understand the mechanism behind an increased ability to sense and avoid high temperatures, studies on heat receptors are needed. An upregulated expression of the heat receptor TrpA1 for example was found in *D. melanogaster* selected at high temperatures^32^. This receptor is also important for heat avoidance in mosquitoes such as *Ae. aegypti*^33^ and may therefore also be upregulated in our warm evolved mosquitoes. In addition to TrpA1, Ir21a and the co-receptor Ir93a also play a role in heat detection^34^. Alternatively, mosquitoes that evolved at lower temperatures might cope better with brief increases in temperatures through an enhanced expression of heat shock proteins. A similar mechanism has been reported for *Drosophila buzzatii* with populations adapted to lower temperatures expressing more heat shock proteins immediately after a heat shock compared to populations that had adapted to warmer temperatures^35^.

Changes in the expressions of these receptors and proteins may be due to epigenetics (i.e., plasticity) when the effect is short-term, as in acclimation, or due to genetic changes after long-term evolution. As both short- and long-term effects were evident in our study, however in opposite directions, the relative contributions of epigenetics and genetics should be investigated in follow-up studies. Moreover, it is important to note that the mosquitoes used in this study originate from a well-established laboratory colony and potentially lack certain traits observed in wild mosquito populations. Hence, similar studies should be repeated using wild or freshly caught populations to ensure the broader applicability of our findings. Nevertheless, our research demonstrates that under controlled laboratory conditions, mosquitoes exhibit significant potential for rapid thermal adaptation which also influences their thermal preference.

### Acclimation effect

The behaviour of mosquitoes acclimated to the higher temperature in the last generation was opposite to the behaviour of those evolved at the warm temperature as they were more abundant in the warmer zones. Mosquitoes reared at higher temperatures might benefit from a plastic increase in their upper thermal limit (T_max_), thus expressing slightly higher tolerance to warm temperatures. In support of this interpretation, a study of *Culex pipiens* showed a 1.5 °C increase in the upper critical (lethal) temperature when mosquitoes were acclimated to higher temperatures^36^. Together with a short-term increase in expression of heat shock proteins^35^, this might explain the preference and increased tolerance of higher temperatures of our warm-acclimated *Ae. aegypti*.

### Flying behaviour

Many physiological processes are facilitated at higher temperatures to become less energy consuming^37^. Because flying requires considerable energy^38,39^, mosquitoes should prefer the warmer zone during this movement type to be more energy efficient. Indeed, mosquitoes in our study spent more time flying in the warmest zone compared to the cooler zones. This preference for warmer temperature zones was modulated both genetically by experimental evolution and plastically by acclimation, although evolution had an overall stronger impact.

Flight velocity was lower for the warm-acclimated mosquitoes, though only in the warmest zone. This effect might be explained by the reduced body sizes of insects acclimated at higher temperature (Table S3). The relationship between body size and temperature, known as the temperature–size (T–S) response^40^. Typically, this response is negative, meaning organisms tend to be smaller when reared in warmer conditions. This pattern is often referred to as the temperature-size rule (TSR). The explanations for this phenomenon range from physiological constraints, such as limited oxygen availability and temperature-dependent resource allocation, to adaptive strategies driven by factors like increased fecundity or shorter development time in response to higher mortality in warm conditions^41^.

### Cold or hot traps

Our thermal gradient setup showed an accumulation of mosquitoes at both the warm and the cool side of the apparatus, suggesting a heat or a cold trap respectively. However, a previous study with the same setup showed that there were no such heat or cold traps^16^, as the insects’ mobility was not impaired at the chosen temperatures, even though such accumulations at the edges are generally expected and found for flying insects in a closed setup^26^.

### Climate change and model adjustment

There are three primary mechanisms used by mosquitoes to respond to warming temperatures^20^. One is *behavioural tracking* of suitable temperatures through range shifts and microhabitat selection. This was shown in previous thermal preference experiments with mosquitoes^15–17,24^. The second mechanism is avoidance or temporary coping with stressful temperatures through *phenotypic plasticity* and the third, more long-term response would be modulating thermal behaviour through *evolutionary adaptation.* The phenotypic plasticity of *Ae. aegypti* mosquitoes was shown in this study in form of short-term acclimatisation over one generation, whereas the evolutionary adaptation has been shown in the form of experimental evolution over 20 generations.

In light of the expected climate warming, evolutionary changes in microhabitat selection over multiple generations has been documented in a variety of species^42^, but not yet for disease vectors. Insect vectors such as mosquitoes are posing a high burden on human and animal health. Evolutionary adaptation of their thermal preference is especially important as it affects their capacity as a vector of pathogens. The life cycle, behaviour, vector competence and physiology of the transmitted pathogens all highly depend on temperature^2^, which is therefore an important element in vector-borne disease models. These models predict that temperatures in certain (tropical) areas may exceed the mosquitoes’ thermal optima and limits, and therefore actually reduce the risk of disease^14,43^. However, these predictions might underestimate risks, since mosquitoes can behaviourally thermoregulate in the short term and evolve their thermal preference, as shown in this study. Thus, areas in which future temperatures are expected to become too high for mosquitoes might remain suitable if the vectors can adapt their thermal behaviour.

## Material and methods

### Mosquito maintenance

The mosquitoes used in this study originated from the UGAL strain of *Ae. aegypti* (obtained from Patrick Guérin, University of Neuchâtel^44^; which had been maintained for many years at 26.5 ± 0.5 °C and 75 ± 5% humidity with a 12:12 h dark:light cycle^45^. To induce hatching of the eggs they were first put into deionised water for one hour and then hatching was facilitated by low pressure for 30 min by applying a vacuum. Thereafter 200 larvae were placed in plastic trays (35 × 21 × 4 cm^3^) filled with 800 ml of deionized water. Larvae were fed with powdered Tetramin baby fish food (Tetra GmbH, Melle, Germany) suspended in ddH_2_O water (0.06 mg/larvae on the day of hatching, 0.08 at day 1 [d1], 0.16 at d2, 0.32 at d3, 0.64 at d4, 0.32 per day for older larvae)^46^. Pupae were collected and transferred into 300 ml plastic cups containing 100 ml deionized water, which were placed into a 21 × 21 × 21 cm^3^ plexiglass cage. Adults were supplied with a 6% sucrose solution and were given the opportunity to take a blood meal on AK’s arm for seven minutes once early (age: 7 days) and once late in life (age: 20 days). Forty-eight hours after the blood meal, they were allowed to lay eggs onto a filter paper lining the walls of plastic cups (300 ml) filled with 100 ml deionized water. Five days after the blood meal filter papers were collected, dried and kept in the dark at room temperature until used for the next generation.

### Mosquito experimental evolution

**Figure 2:**
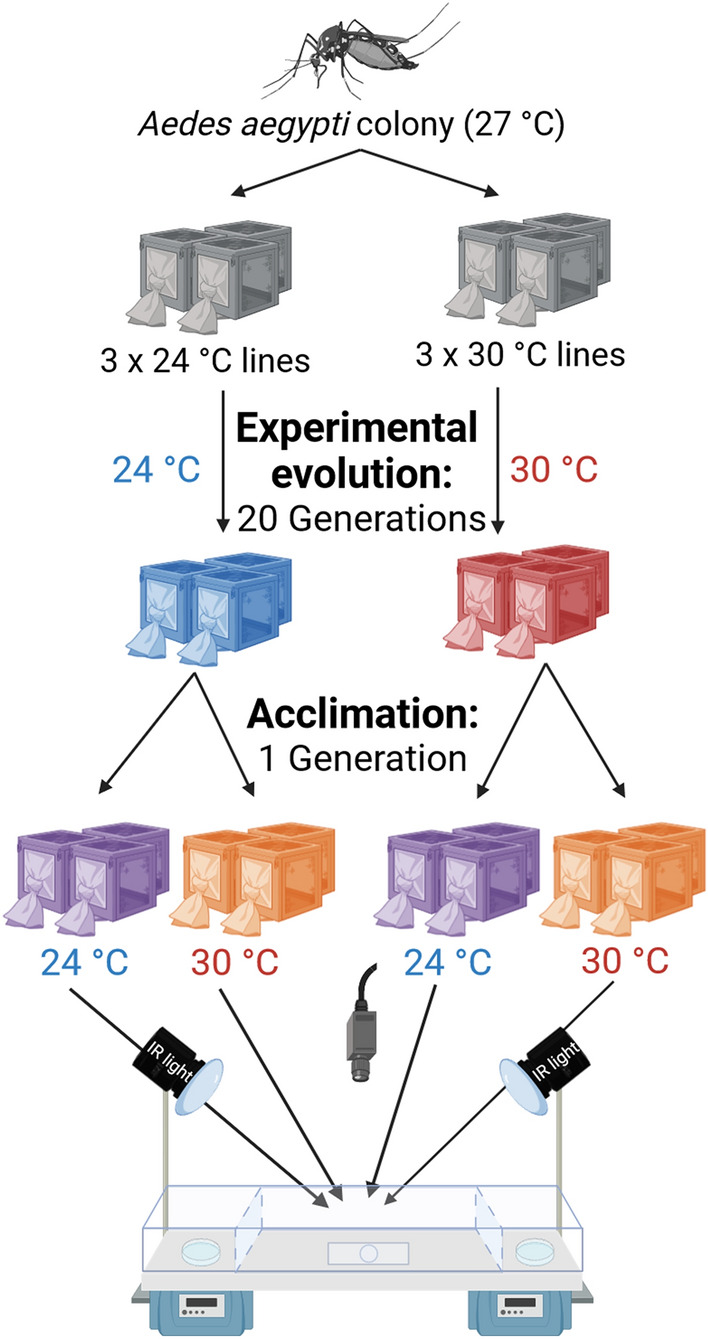
Scheme of experimental evolution and acclimation of mosquitoes at different temperatures. The original colony was split into four experimental temperatures, and three cohorts were raised per temperature. Only the cohorts evolved at the most extreme temperatures (24 °C, blue; 30 °C, red) were used in this study. Eggs of mosquitoes from these cohorts were then acclimated at 24 °C (purple) or 30 °C (orange). Thermal preference was tested in a full factorial design in a thermal gradient setup. Created with BioRender.com.

Three replicate cohorts (groups) were adapted at 24 ± 0.3 °C and three at 30 ± 0.3 °C (Fig. 2). Triplicates were created for each temperature to avoid any population effect unrelated to the experimental treatment^47^. The temperature was increased or decreased by 0.7 °C every generation until the target temperature regime was reached. Relative humidity and dark:light cycle were not changed. Larvae and adults were kept at the same temperature throughout the experimental evolution. Once the target temperature was reached, each cohort was reared for 20 more generations at the target temperature and used in the experiments. Eggs from all cohorts were dried out at their current evolutionary temperature for 48h before being stored at room temperature in the dark in between generations. All cohorts were hatched simultaneously for each subsequent generation.

### Mosquito acclimation

Larvae of the 20^th^ generation of the adapted cohorts were reared individually in 12-well plates at either 24 ± 0.5 °C or 30 ± 0.5 °C (relative humidity and light regime as above). Each well contained 3 ml of deionized water and 0.06 mg Tetramin baby fish food suspended in 100 µl water. The feeding schedule was the same as in the colony. Pupae of each cohort and rearing temperature were moved to a Petri dish with some water, and the Petri dish was placed in a large plastic cup covered with netting. Adults had access to 5% glucose solution for seven days until the experiment.

### Thermal gradient setup and experimental design

Details of the thermal gradient setup were described before^15^. Briefly, it consisted of two thermal regulators (AHP-1200CPV, ThermoElectric Cooling America Corporation, Chicago, USA) connected by an aluminium plate (91 × 30 × 2.5 cm^3^, TGB-5030, ThermoElectric) and covered with a custom-made, transparent Plexiglas box (80 × 30 × 4 cm^3^). Mosquitoes were filmed with a camera (1/1.8″ CMOS sensor, 1280 × 1024, 60 fps, acA1300-60gm; Basler, Ahrensburg, Germany) placed above this setup and equipped with an infrared light filter to exclude any changes in lighting in the visible spectrum. Four infrared lights (VAR2-i2-1 short range infra-red illuminator; Raytec, Ashington, UK) were used to light the setup indirectly (minimizing shadow casting). Experiments were performed in a room without windows. Two LED strips ensured uniform illumination. The gradient was chosen to span 5 ± 0.5 °C below and 5 ± 0.5 °C above the adaptation temperatures. The gradient was applied one hour before experiments started. Temperatures were recorded every five seconds by three dataloggers (MSR 145, MSR Electronics GmbH, Seuzach, Switzerland) at each end (20 ± 0.5 °C or 35 ± 0.5 °C) and the middle (27.5 ± 0.5 °C) of the gradient to ensure a linear distribution of temperature. The cool and warm sides were switched between experiments to exclude side effects. Salt solutions were used to keep the humidity stable across the gradient as described in^15^. The gradient setup was only touched with gloves to prevent contamination with human odours and cleaned each day with 30% ethanol.

We followed a full factorial design. One trial consisted of 10 to 15 female mosquitoes of one of the six cohorts reared at one of the two temperatures (24 °C and 30 °C), yielding 12 trials per block. These trials were repeated six times for each cohort over six weeks. The order of the trials was randomized within each block. In each trial the mosquitoes were released in the middle of the gradient and their activity recorded for 15 min.

### Tracking and video analysis

Videos were analysed using the EthoVision video analysis software (XT 14, Noldus Information Technology, Wageningen, the Netherlands). This program distinguishes between background and darker individuals to track the movement of individual mosquitoes. Tracking of position over time allows to calculate information such as velocity, distance, and duration of various locomotor behaviours of individuals^48^. Video analysis was performed as described previously^16^. To get the background image, one of the first 10 frames without mosquitoes was used, and the recording of movement was only started after all individuals were present in the frame. The arena was subdivided into five temperature zones of 12 × 30 cm (zones 1–5; 20–35 ± 0.5 °C), with zone 1 always being the coolest one. Detection settings were checked for each video but only adjusted when needed, and each tracking record lasted 15 min. The following parameters were analysed: the cumulative (total) duration spent flying, walking, or resting per individual, the mean distances covered, and the mean velocity. Distinguishing between flying (> 1.5 cm/s), walking, and resting (< 0.1 cm/s) was done according to the criteria described by^16,49^.

### Statistical analysis

For our analysis, four datasets were established: one with all data, plus one each with only flying, resting, and walking data. For each of these data sets the cumulative duration in each zone [s] and the mean velocity [cm/s] were analysed. Four trials that included fewer than ten mosquitoes, in one treatment, were excluded from analysis. Those were repeated in four additional experimental blocks including all treatments.

For model selection, the distribution of each of these variables was checked. We used a linear mixed model for log_10_(mean velocity), to reach normality of the residuals, with experimental evolution and acclimation temperature as well as temperature zone as factorial explanatory variables, including all interactions. Experimental run nested in day, cohorts nested in experimental temperature, and the individual were additional random factors. The model with all movement types additionally included movement type as an explanatory variable. Assumptions of the model were assessed visually using the autofit function from the ggfortify package in R^50^.

Since mosquitoes that were acclimated to 24 °C were tracked in average 4% longer than those acclimated to 30 °C, cumulative duration in the zones was transformed into relative durations and, therefore, analysed with a generalized linear mixed model with binomial family and logit link.

Relative durations were thus the response variable of these GLMMs. Experimental evolution and acclimation temperatures as well as temperature zones all as factors, were the explanatory variables. Random effects included experimental run nested in day, cohorts nested in experimental temperature, and the individual.

For all models, multiple post-hoc pairwise comparisons of means with Tukey contrasts were performed for each significant interaction. All analyses were performed in R v. 4.1.0^51^. The lme4 package v. 1.1-31 was used for modelling^52^, the ggplot2 package v.3.4.0 for visualization^53^, and the dplyr package v.2.2.1 for data clean-up^54^.

### Supplementary Information

Supplementary Information.

## Data Availability

Code and data can be found under: 10.5281/zenodo.10228932.
