# Supplementary material for: Unexpected behavioural adaptation of yellow fever mosquitoes in response to high temperatures
Source: Sci Rep. 2024 Feb 13;14:3659. doi: 10.1038/s41598-024-54374-5 (PMC10864274; doi:10.1038/s41598-024-54374-5)
Supplement: Supplementary file 1 — Supplementary Information. [file 41598_2024_54374_MOESM1_ESM.docx]

# Supporting information

# Unexpected behavioural adaptation of yellow fever mosquitoes in response to high temperatures

David O. H. Hug^1#^, Alida Kropf^2#^, Marine Amann^2^, Jacob C. Koella^2^, Niels O. Verhulst^1*^

^1^National Centre for Vector Entomology, Institute of Parasitology, Vetsuisse and Medical Faculty, University of Zürich, Zürich, Switzerland

^2^Laboratory of Ecology and Epidemiology of Parasites, Institute of Biology, University of Neuchâtel, Neuchâtel, Switzerland

^#^These authors contributed equally


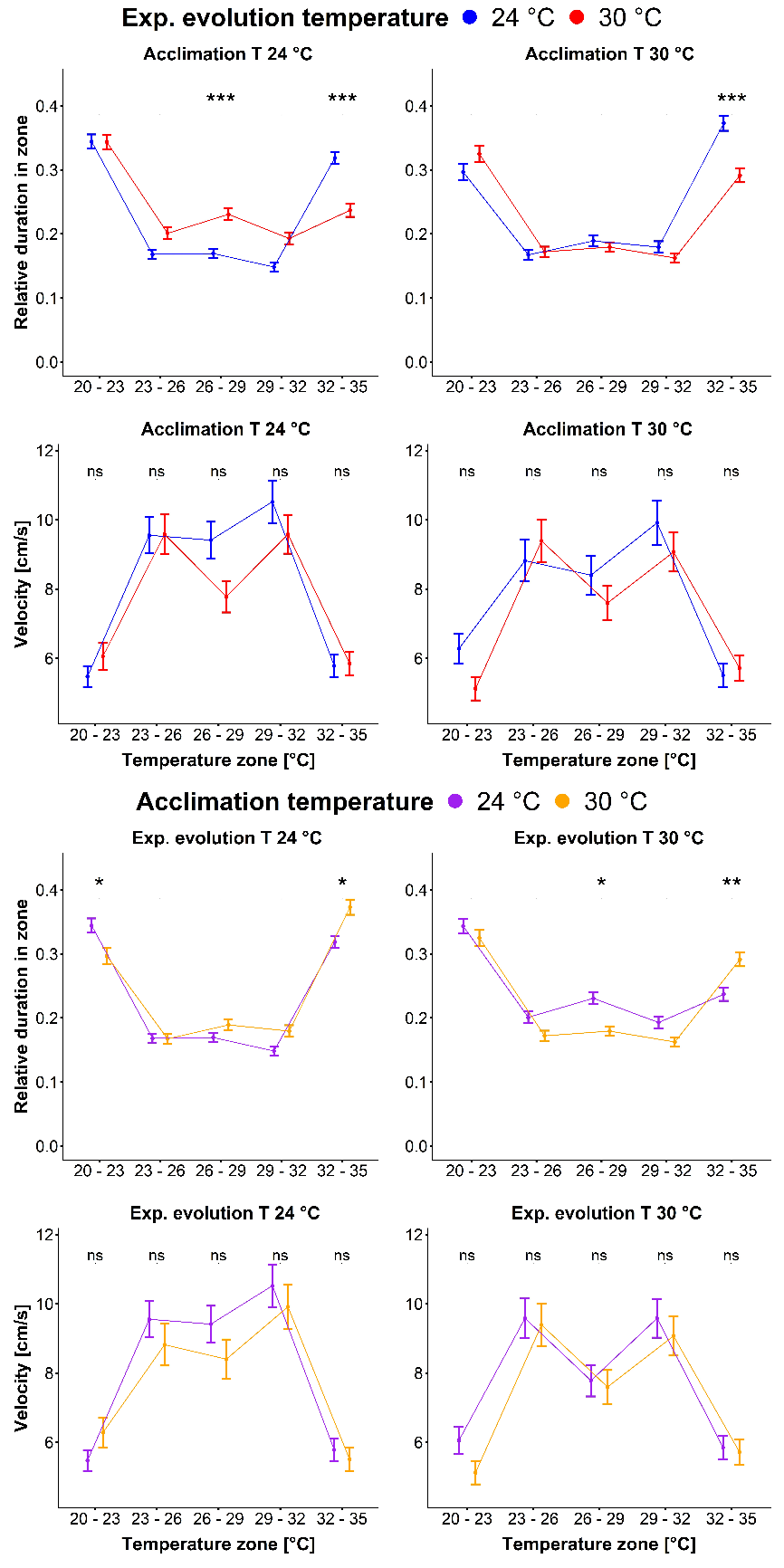


**Figure S1. Relative duration of stay of the mosquitoes in the different temperature zones and their velocity therein.** The top block of four graphs indicates differences in experimental evolution temperatures of 24 °C (blue) and 30 °C (red) for acclimation temperatures of 24 °C (left) and 30 °C (right). Where the top two show differences in relative duration of stay of the experimental evolution cohorts and the lower two show the velocity for the corresponding mosquito groups. The lower block of four graphs indicates differences in acclimation temperatures of 24 °C (purple) and 30 °C (orange) for experimental evolution temperatures of 24 °C (left) and 30 °C (right). Where the top two show differences in relative duration of stay of the experimental evolution cohorts and the lower two show the velocity for the corresponding mosquito groups. Shown data are means over all individuals in the group and the standard error. Significance levels (* = P < 0.05, ** = P < 0.01, *** = P < 0.001) are indicated between treatments within one zone (Tukey; 95 % confidence level). N = 1024 (527 acclimated at 24°C; 497 at 30°C; 505 evolved at 24°C and 519 at 30°C).


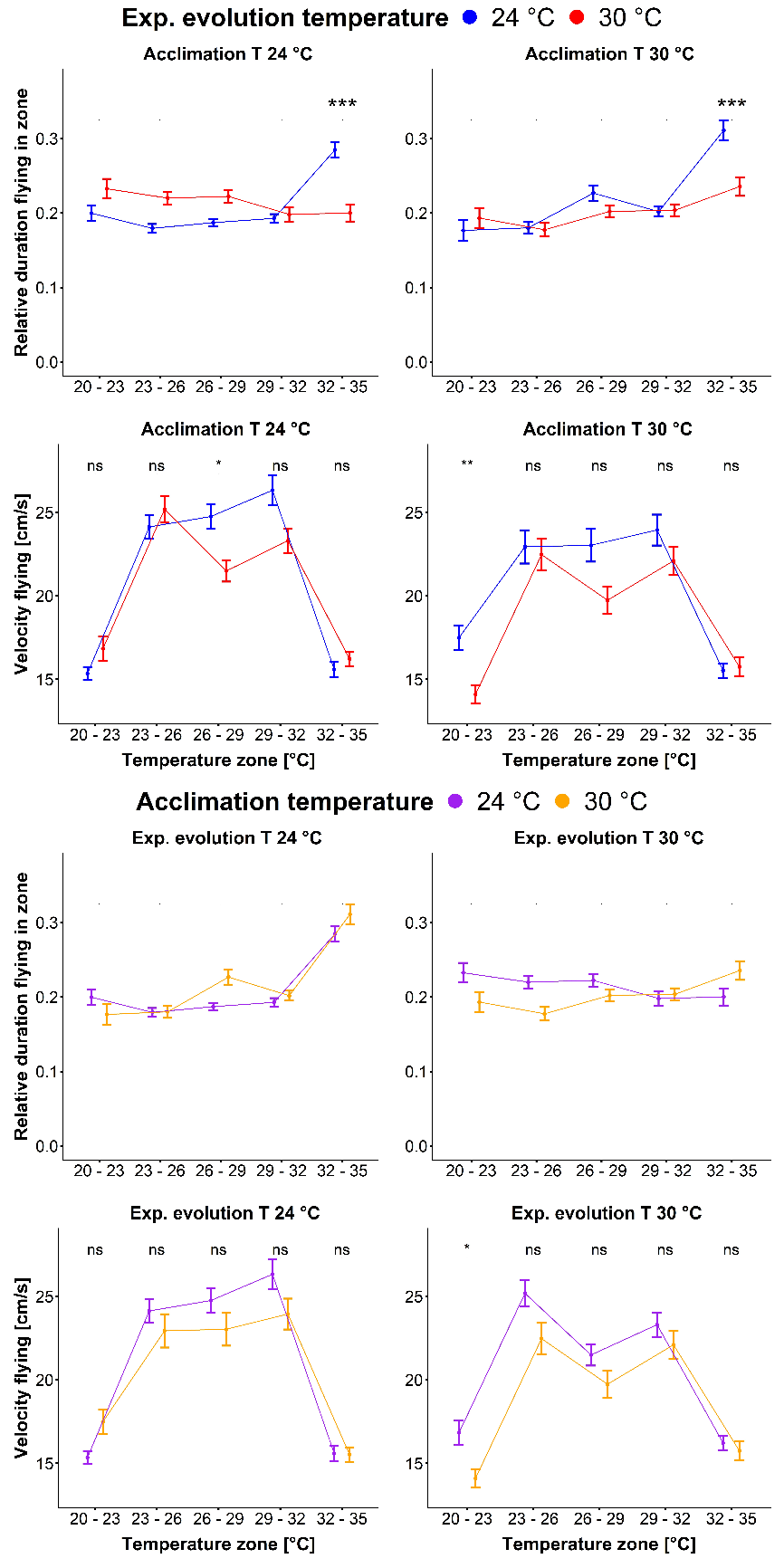


**Figure S2. Relative duration of flying of the mosquitoes in the different temperature zones and their velocity therein.** The top block of four graphs indicates differences in experimental evolution temperatures of 24 °C (blue) and 30 °C (red) for acclimation temperatures of 24 °C (left) and 30 °C (right). Where the top two show differences in relative duration of flying of the experimental evolution cohorts and the lower two show the velocity of flying for the corresponding mosquito groups. The lower block of four graphs indicates differences in acclimation temperatures of 24 °C (purple) and 30 °C (orange) for experimental evolution temperatures of 24 °C (left) and 30 °C (right). Where the top two show differences in relative duration of stay of the experimental evolution cohorts and the lower two show the velocity of flying for the corresponding mosquito groups. Shown data are means over all individuals in the group and the standard error. Significance levels (* = P < 0.05, ** = P < 0.01, *** = P < 0.001) are indicated between treatments within one zone (Tukey; 95 % confidence level). N = 1024 (527 acclimated at 24°C; 497 at 30°C; 505 evolved at 24°C and 519 at 30°C).


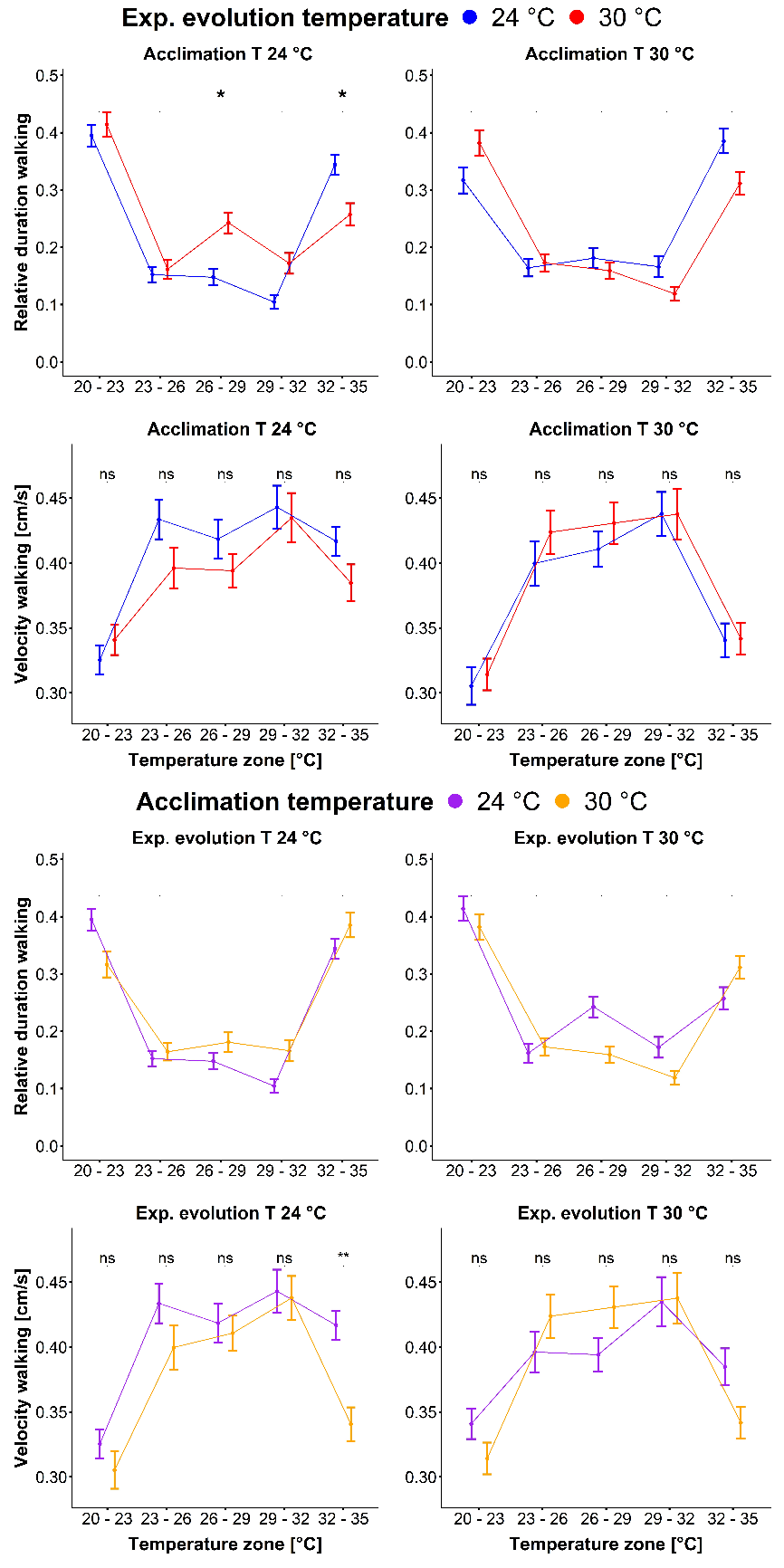


**Figure S3. Relative duration of walking of the mosquitoes in the different temperature zones and their velocity therein.** The top block of four graphs indicates differences in experimental evolution temperatures of 24 °C (blue) and 30 °C (red) for acclimation temperatures of 24 °C (left) and 30 °C (right). Where the top two show differences in relative duration of walking of the experimental evolution cohorts and the lower two show the velocity of walking for the corresponding mosquito groups. The lower block of four graphs indicates differences in acclimation temperatures of 24 °C (purple) and 30 °C (orange) for experimental evolution temperatures of 24 °C (left) and 30 °C (right). Where the top two show differences in relative duration of waling of the experimental evolution cohorts and the lower two show the velocity of walking for the corresponding mosquito groups. Shown data are means over all individuals in the group and the standard error. Significance levels (* = P < 0.05, ** = P < 0.01, *** = P < 0.001) are indicated between treatments within one zone (Tukey; 95 % confidence level). N = 1024 (527 acclimated at 24°C; 497 at 30°C; 505 evolved at 24°C and 519 at 30°C).


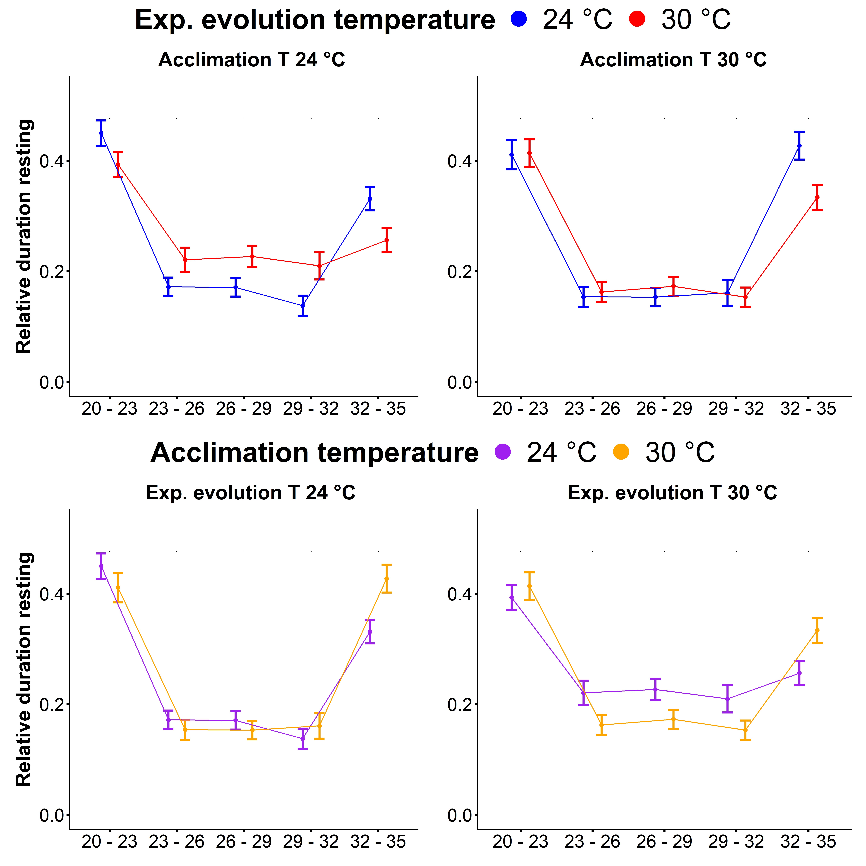


**Figure S4. Relative duration of resting of the mosquitoes in the different temperature zones.** All graphs show differences in relative duration of resting of the experimental evolution cohorts in a certain temperature zone. The top two graphs indicate differences in experimental evolution temperatures of 24 °C (blue) and 30 °C (red) for acclimation temperatures of 24 °C (left) and 30 °C (right). The lower two graphs indicate differences in acclimation temperatures of 24 °C (purple) and 30 °C (orange) for experimental evolution temperatures of 24 °C (left) and 30 °C (right). Shown data are means over all individuals in the group and the standard error. Significance levels (* = P < 0.05, ** = P < 0.01, *** = P < 0.001) indicated between treatments within one zone (Tukey; 95 % confidence level). N = 1024 (527 acclimated at 24°C; 497 at 30°C; 505 evolved at 24°C and 519 at 30°C).

**Table S1. Generalised linear model of relative abundance as ANOVA table.**

|  | LR Chisq | Df | Pr(>Chisq) |
| --- | --- | --- | --- |
| All movement types |  |  |  |
| evolution_temp | 7.705 | 1 | 0.006 |
| acclimation_temp | 0.232 | 1 | 0.630 |
| temp_zone | 1030.946 | 4 | > 0.001 |
| day | 88.961 | 15 | > 0.001 |
| run | 24.500 | 7 | 0.001 |
| movement_type | 89.952 | 2 | > 0.001 |
| evolution_temp:acclimation_temp | 7.984 | 1 | 0.005 |
| evolution_temp:temp_zone | 86.952 | 4 | > 0.001 |
| acclimation_temp:temp_zone | 43.267 | 4 | > 0.001 |
| evolution_temp:acclimation_temp:temp_zone | 27.210 | 4 | > 0.001 |
| Flying |  |  |  |
| evolution_temp | 3.474 | 1 | 0.062 |
| acclimation_temp | 0.682 | 1 | 0.409 |
| temp_zone | 115.288 | 4 | > 0.001 |
| day | 39.781 | 15 | > 0.001 |
| run | 9.202 | 7 | 0.239 |
| evolution_temp:acclimation_temp | 6.355 | 1 | 0.012 |
| evolution_temp:temp_zone | 68.393 | 4 | > 0.001 |
| acclimation_temp:temp_zone | 23.596 | 4 | > 0.001 |
| evolution_temp:acclimation_temp:temp_zone | 9.646 | 4 | 0.047 |
| Walking |  |  |  |
| evolution_temp | 0.772 | 1 | 0.380 |
| acclimation_temp | 0.042 | 1 | 0.837 |
| temp_zone | 573.067 | 4 | > 0.001 |
| day | 26.058 | 15 | 0.037 |
| run | 9.712 | 7 | 0.205 |
| evolution_temp:acclimation_temp | 4.176 | 1 | 0.041 |
| evolution_temp:temp_zone | 27.915 | 4 | > 0.001 |
| acclimation_temp:temp_zone | 15.711 | 4 | 0.003 |
| evolution_temp:acclimation_temp:temp_zone | 25.655 | 4 | > 0.001 |
| Resting |  |  |  |
| evolution_temp | 5.194 | 1 | 0.023 |
| acclimation_temp | 0.116 | 1 | 0.733 |
| temp_zone | 452.546 | 4 | > 0.001 |
| day | 41.027 | 15 | > 0.001 |
| run | 8.169 | 7 | 0.318 |
| evolution_temp:acclimation_temp | 0.759 | 1 | 0.383 |
| evolution_temp:temp_zone | 24.538 | 4 | > 0.001 |
| acclimation_temp:temp_zone | 23.046 | 4 | > 0.001 |
| evolution_temp:acclimation_temp:temp_zone | 7.065 | 4 | 0.132 |

**Table S2. Linear model of log_10_ velocity as ANOVA table.**

|  | Df | F value | Pr(>F) |
| --- | --- | --- | --- |
| All movement types |  |  |  |
| evolution_temp | 1 | 12.234 | > 0.001 |
| acclimation_temp | 1 | 4.211 | 0.040 |
| temp_zone | 4 | 377.846 | > 0.001 |
| day | 15 | 22.741 | > 0.001 |
| run | 7 | 10.518 | > 0.001 |
| movement_type | 2 | 163715.795 | > 0.001 |
| evolution_temp:acclimation_temp | 1 | 0.063 | 0.802 |
| evolution_temp:temp_zone | 4 | 0.639 | 0.635 |
| acclimation_temp:temp_zone | 4 | 2.039 | 0.086 |
| evolution_temp:acclimation_temp:temp_zone | 4 | 3.459 | 0.008 |
| residuals | 12284 |  |  |
| Flying |  |  |  |
| evolution_temp | 1 | 19.724 | > 0.001 |
| acclimation_temp | 1 | 39.330 | > 0.001 |
| temp_zone | 4 | 150.144 | > 0.001 |
| day | 15 | 35.772 | > 0.001 |
| run | 7 | 11.697 | > 0.001 |
| evolution_temp:acclimation_temp | 1 | 2.560 | 0.110 |
| evolution_temp:temp_zone | 4 | 4.515 | 0.001 |
| acclimation_temp:temp_zone | 4 | 1.470 | 0.208 |
| evolution_temp:acclimation_temp:temp_zone | 4 | 2.619 | 0.033 |
| residuals | 4540 |  |  |
| Walking |  |  |  |
| evolution_temp | 1 | 2.027 | 0.155 |
| acclimation_temp | 1 | 7.547 | 0.006 |
| temp_zone | 4 | 43.693 | > 0.001 |
| day | 15 | 20.275 | > 0.001 |
| run | 7 | 2.915 | 0.005 |
| evolution_temp:acclimation_temp | 1 | 1.827 | 0.177 |
| evolution_temp:temp_zone | 4 | 1.904 | 0.107 |
| acclimation_temp:temp_zone | 4 | 6.093 | > 0.001 |
| evolution_temp:acclimation_temp:temp_zone | 4 | 1.341 | 0.252 |
| residuals | 4074 |  |  |
| Resting |  |  |  |
| evolution_temp | 1 | 6.852 | 0.009 |
| acclimation_temp | 1 | 0.191 | 0.662 |
| temp_zone | 4 | 51.361 | > 0.001 |
| day | 15 | 11.680 | > 0.001 |
| run | 7 | 2.697 | 0.009 |
| evolution_temp:acclimation_temp | 1 | 0.039 | 0.843 |
| evolution_temp:temp_zone | 4 | 0.784 | 0.536 |
| acclimation_temp:temp_zone | 4 | 3.382 | 0.009 |
| evolution_temp:acclimation_temp:temp_zone | 4 | 7.359 | > 0.001 |
| residuals | 3588 |  |  |

**Table S3. Wing length (mm) of Ae. aegypti mosquitoes evolved and acclimated at different temperatures.** Wing lengths are in mm ± standard deviation. N = 31-49 for each cohort. Mosquitoes experimentally evolved at 30 °C where slightly but not significantly smaller than mosquitoes evolved at 24 °C (t-test, t_497.46_ = 1.97, P = 0.050). Mosquitoes acclimated at 30 °C were significantly smaller than mosquitoes acclimated at 24 °C (t-test, t_501.37_ = 30.10, P < 0.001).

|  |  | Exp. evolution 24°C | | | |  | Exp. evolution 30°C | | | |
| --- | --- | --- | --- | --- | --- | --- | --- | --- | --- | --- |
|  |  | Cohort 1 | Cohort 2 | Cohort 3 | Mean ± SD |  | Cohort 10 | Cohort 11 | Cohort 12 | Mean ± SD |
| Acclimation 24°C |  | 3.19 ± 0.13 | 3.15 ± 0.12 | 3.18 ± 0.13 | 3.17 ± 0.13 |  | 3.07 ± 0.22 | 3.12 ± 0.14 | 3.07 ± 0.12 | 3.09 ± 0.16 |
| Acclimation 30°C |  | 2.73 ± 0.14 | 2.75 ± 0.13 | 2.75 ± 0.10 | 2.75 ± 0.12 |  | 2.75 ± 0.16 | 2.79 ± 0.14 | 2.74 ± 0.13 | 2.76 ± 0.14 |
| Mean ± SD |  | 2.99 ± 0.26 | 2.91 ± 0.23 | 2.97 ± 0.24 | 2.96 ± 0.25 |  | 2.89 ± 0.24 | 2.95 ± 0.22 | 2.91 ± 0.22 | 2.92 ± 0.22 |
